# Increasing Profitability of Ethanol Photoreforming by Simultaneous Production of H_2_ and Acetal

**DOI:** 10.1002/gch2.202300078

**Published:** 2023-07-06

**Authors:** Oleksandr Savateev, Vitaliy Shvalagin, Junwang Tang

**Affiliations:** ^1^ Colloid Chemistry Department Max Planck Institute of colloids and Interfaces Am Muehlenberg 1 14476 Potsdam Germany; ^2^ Pisarzhevskii Institute of Physical Chemistry of the NAS of Ukraine Prospect Nauky, 31 Kyiv 03028 Ukraine; ^3^ Department of Chemical Engineering University College London Torrington Place London WC1E 7JE UK; ^4^ Industrial Catalysis Center Department of Chemical Engineering Tsinghua University Beijing 100084 China

**Keywords:** hydrogen, organic synthesis, photocatalysis

## Abstract

Often, H_2_ is produced photocatalytically at the expense of sacrificial agents. When a sacrificial agent is selectively oxidized, this allows coupling of H_2_ production with synthesis of value‐added organic compounds. Herein, it is argued that the conversion of bioethanol into 1,1‐diethoxyethane with simultaneous H_2_ production increases the economic viability of photocatalysis and suggests a semiconductor material that is the most relevant for this purpose.

## Graphical abstract

Ethanol is converted into value‐added products, 1,1‐diethoxyethane and H_2_, by employing heterogeneous photocatalysis and acid catalysis. This reaction is promising strategy to transfer photocatalysis from laboratory to industrial scale.

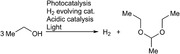

## 1 Introduction

Society is urgently aiming for the decarbonization of the chemical industry. In these endeavors, green hydrogen, which is produced using only abundant sources and renewable energy, is seen as a primary energy carrier. Electrolysis of water is a mature technology. In 2021, global production of H_2_ stood at 75 Mt per year, among which 1% could be regarded as green hydrogen generated upon water electrolysis using renewable energy.^[^ ^1^ ^]^ More recent, but the rapidly developing is photocatalytic splitting of water over metal oxide semiconductors.^[^ ^2^ ^]^ This approach intends to use solar light directly to drive complete water splitting into H_2_ and O_2_, complementary the conversion of solar light into electricity. In both electrolysis and photocatalysis, water is used as the reagent, which without doubt is the most abundant source of H_2_ on the planet. However, the production of 1 mole (2 g) of H_2_ is accompanied by 0.5 moles (16 g) of O_2_. While H_2_ is a high‐value product, given the abundance of O_2_ in Earth's atmosphere, the latter has limited value.

## 2 Discussion

**Figure 1:**
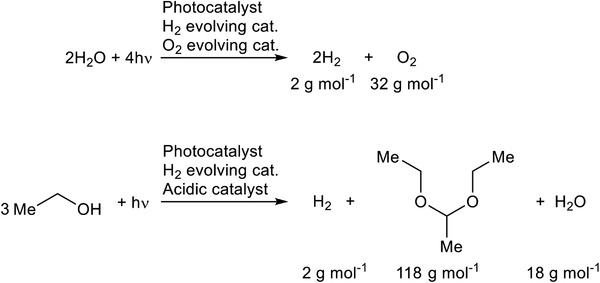
Photocatalytic water splitting and photoreforming of ethanol into H_2_ and 1,1‐diethoxyethane.

In order to make photocatalytic or electrocatalytic production of H_2_ more economically viable, it is imperative to couple its production with other value‐added molecules.^[^ ^3^ ^]^ One alternative is the photoreforming of bioethanol into H_2_ and high‐value chemical, e g., 1,1‐diethoxyethane (**Figure** **1**).^[^ ^4^ ^]^ The advantage of this reaction is the synthesis of 1 g of H_2_ along with 118 g of organic molecule that could be applied such as an oxygenated additive for diesel,^[^ ^5^ ^]^ solvent in organic synthesis,^[^ ^6^, ^7^ ^]^ solvent for methyllithium,^[^ ^8^ ^]^ reagent to introduce protecting group in organic synthesis,^[^ ^9^ ^]^ fragrance.^[^ ^10^ ^]^

**Figure 2:**
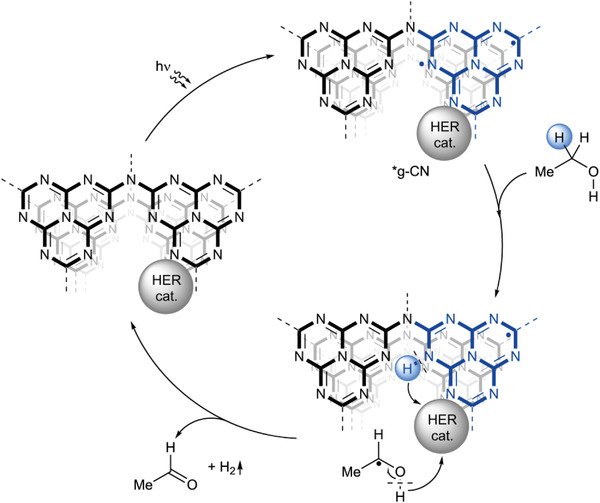
Concept of ethanol dehydrogenation mechanism over a photocatalytic system composed of heptazine‐based g‐CN and H_2_ evolving catalyst.

Assuming photocatalytic conversion of ethanol into acetal is the target reaction, what photocatalytic system would be optimal in terms of selectivity and efficiency of solar energy utilization? Taking into account a possible mechanism of alcohols oxidation,^[^ ^11^ ^]^ the photocatalyst structure and potential of the valence band must favor abstracting hydrogen atom from *α*‐position to the hydroxyl‐group to trigger dehydrogenation of ethanol (**Figure** **2**). These requirements are met in heptazine‐based graphitic carbon nitrides (g‐CN). These are 2D materials composed of electron‐deficient heterocycles that are rich in (sp^2^)N atoms. Upon excitation with light, the conjugated electron deficient structure provides an environment for temporary storage of electrons, while the edge facet on the nanocrystals terminated by (sp^2^)N atoms serves as the site where protons are stored.^[^ ^12^ ^]^ Overall, carbon nitride photocatalyst via excited state proton‐coupled electron transfer cleaves C–H bond in ethanol.

In particular, excited state of covalent graphitic carbon nitride, such as mesoporous graphitic carbon nitride, is capable of cleaving bonds that are characterized by bonds dissociation free energy (BDFE) approx. 418 kJ mol^−1^.^[^ ^12^ ^]^ Ionic carbon nitrides,^[^ ^13^ ^]^ such as potassium poly(heptazine imide) (K‐PHI), due to more positive potential of the valence band of +2.3 V versus NHE and basic character of the surface (apparent p*K*_a_ of the conjugated acid is approx. 7) are able to cleave even stronger bonds with BDFE ≥ 490 kJ mol^−1^.^[^ ^12^ ^]^ Given that C–H BDFE in ethanol in *α*‐position to hydroxyl group is approx. 377 kJ mol^−1^, both covalent and ionic carbon nitrides are able to trigger this initial step of ethanol photoreforming. In fact, this is supported by plenty of experimental data, in which graphitic carbon nitrides produce H_2_ in the presence of sacrificial electron donors, such as alcohols.^[^ ^14^, ^15^ ^]^

More negative potential of the conduction band creates a stronger driving force for hydrogen evolution reaction (HER). However, employment of H_2_‐evolving co‐catalyst, Pt or Pd nanoparticles,^[^ ^16^ ^]^ molecular molybdenum sulfide complex,^[^ ^17^ ^]^ Ni‐molecular complex,^[^ ^18^ ^]^ etc. allows for the conduction band to be only slightly more negative than the standard redox potential of the reaction 2H^+^ + 2e^–^ = H_2_ (*E* ^0^ = 0 V versus normal hydrogen electrode (NHE) at pH = 0). In fact, ionic carbon nitrides, which are characterized by the conduction band potential of –0.1…–0.5 V versus NHE,^[^ ^19^, ^20^ ^]^ demonstrate remarkably high apparent quantum yield (AQY) > 50% at 420 nm in H_2_ production in the presence of triethanolamine (10 vol. %) and photodeposited Pt nanoparticles.^[^ ^21^, ^22^, ^23^ ^]^ These experimental evidences and relatively low (sp^3^)C‐H BDFE value in ethanol offers opportunities for designing carbon nitride semiconductors having less positive potential of the valence band. Overall, assuming surface basicity of such engineered graphitic carbon nitride to be the same as in K‐PHI the potential of the valence band need to be only approx. +1.1 V versus NHE in order for the excited state to be sufficiently energetic to convert ethanol into ketyl radical upon excited state proton‐coupled electron transfer.^[^ ^12^ ^]^ Such carbon nitride would have optical band gap of 1.2‐1.6 eV and therefore onset of absorption at 780–1000 nm.

Ketyl radical is a strong reductant.^[^ ^24^ ^]^ Therefore, it is converted into acetaldehyde upon spontaneous transfer of an electron and a proton to the photocatalyst. Thus, one photon is required to split ethanol molecule into acetaldehyde and H_2_. Conversion of acetaldehyde into 1,1‐diethoxyethane is then catalyzed by acid.^[^ ^4^ ^]^ Given that synthesis of 1,1‐diethoxyethane from acetaldehyde and ethanol is accompanied by formation of water, adding water absorbers allows increasing selectivity towards this product.

The mechanism scheme shown in Figure 2 is based on the photocatalytic system composed of a single semiconductor. Coupling of ionic carbon nitride with other semiconductors in a Z‐scheme would allow for enhanced efficiency of overall solar energy utilization in bioethanol conversion into H_2_ and acetal.

Taking into account only the price of reagents and products (Merck catalogue), synthesis of 1 kg of H_2_ and 72 L of acetal from 87 L of ethanol generates approx. 10 k€. Finding new applications of acetal, a biomass‐derived molecule, among which probably the most appealing is its use as solvent,^[^ ^6^ ^]^ will further increase the viability of ethanol photoreforming into H_2_ and acetal. On the other hand, valorization of acetal into C4‐products via pinacol‐like coupling in the photocatalytic cascade reaction is another promising pathway.^[^ ^25^ ^]^ These molecules potentially could be applied as fuel.

## 3 Conclusion

Overall, the development of cost‐effective photocatalysts, such as those based on g‐CN, which are selective toward the production of value‐added molecules from biomass‐derived compounds with AQY > 50% upon irradiation with photons in the visible range of the electromagnetic spectrum, is a promising strategy to transfer photocatalysis from laboratory to industrial scale.

## Conflict of Interest

A patent WO/2019/081036 has been filed by Max Planck Gesellschaft zur Förderung der Wissenschaften E.V. in which O.S. is listed as a co‐author.
